# Frailty Pathogenesis, Assessment, and Management in Older Adults With COVID-19

**DOI:** 10.3389/fmed.2021.694367

**Published:** 2021-07-06

**Authors:** Quan She, Bo Chen, Wen Liu, Min Li, Weihong Zhao, Jianqing Wu

**Affiliations:** Department of Geriatrics, Jiangsu Provincial Key Laboratory of Geriatrics, First Affiliated Hospital, Nanjing Medical University, Nanjing, China

**Keywords:** COVID-19, frailty, pathogenesis, assessment, management

## Abstract

The 2019 coronavirus disease (COVID-19) is a highly contagious and deadly disease. The elderly people are often accompanied by chronic inflammation and immunodeficiency, showing a frail state. The strength, endurance, and physiological function of the elderly are significantly decreased, and the ability to deal with stress response is weakened. They are the high-risk group that suffering from COVID-19, and rapidly developing to critical illness. Several recent studies suggest that the incidence rate of COVID-19 in elderly patients with frailty is high. Early assessment, detection, and effective intervention of frailty in COVID-19 patients are conducive to significantly improve the quality of life and improve prognosis. However, there are insufficient understanding and standards for the current evaluation methods, pathogenesis and intervention measures for COVID-19 combined with frailty. This study reviews the progress of the research on the potential pathogenesis, evaluation methods and intervention measures of the elderly COVID-19 patients with frailty, which provides a reference for scientific and reasonable comprehensive diagnosis and treatment in clinical.

## Introduction

Novel coronavirus SARS-CoV-2, named “coronavirus disease 2019” (COVID-19) by the World Health Organization (WHO) on February 11, 2020, is one of the highly pathogenic β-coronaviruses. This coronavirus spreading through the respiratory tract to cause pneumonia showed high infectivity and mortality. As of March 1, 2021, the COVID-19 pandemic has spread to 192 countries with over 114.2 million confirmed cases and 2.3 million deaths (1). It is reported that the mortality of COVID-19 rose exponentially with age, from 0.4% among those aged 40–49 years to 3.6% among those aged 60–69 years and 14.8% among those aged >80 years (2). The possible reasons for this phenomenon are changes of physiological structure and function, accompanied by chronic inflammation, immune decline, and decreased anti-stress ability, which are not conducive to the prognosis of COVID-19. In this paper, the epidemiology, potential pathogenesis, evaluation methods, and intervention measures of old COVID-19 patients combined with frailty are reviewed, which can provide reference for scientific and reasonable clinical comprehensive diagnosis and treatment.

## Definition of Frailty

Frailty, which is common in all countries, is a major cause of functional decline and early death in the elderly (3, 4). This syndrome is either physical or psychological or a combination of both, and is a dynamic process that can improve or worsen over time. In 2013, a consensus group defined frailty as “A medical syndrome with multiple causes and contributors that is characterized by diminished strength, endurance, and reduced physiologic function that increases an individual's vulnerability for developing increased dependency and/or death,” consisting of delegates from six major international, European, and US societies (5). There are two principal models. In Fried's phenotype model, frailty is based on three or more components of poor grip strength, slow walking speed, low physical activity, exhaustion, and unintentional weight loss (6). The frailty index, or cumulative deficit model, defines frailty in terms of the accumulation of “deficits” (symptoms, signs, diseases, and disabilities). A frailty index score reflects the proportion of potential deficits present (7). The phenotype model defines frailty in physical terms, whereas the cumulative deficit model uses a broader definition of frailty.

## Epidemiology

Frailty can occur before the age of 65, but the probability of frailty is on the rise in the elderly aged 70 and above (8). Studies suggest that the incidence of frailty reaches 15% in the elderly over 65 years old and more than 25% in people over 85 years old (9). Incidence rate in women is higher than in men (10, 11). An important reason suggested is that old women with frailty have more abdominal fat than old men (12). Abdominal adiposity is associated with systemic inflammation by mediating its link with metabolic syndromes, which are important markers of oxidative stress and result in skeletal muscle damage and low grip strength (12). This factor might be a core mechanism leading to sex-associated frailty. Besides, lower education level, lower income, lower socioeconomic status, and minority are all positively related to the frailty (13, 14). These persons tend to be frailer, lonelier, and more isolated than others. A prospective study demonstrated high levels of loneliness were associated with increased risk of physical frailty (15).

One study enrolled 1,564 COVID-19 patients with the median age of 74 years old. According to the clinical frailty scale (CFS), the incidence of frailty (CFS score 5–9) was 49.4%. Results showed that frail patients had both an increased risk of mortality and longer duration of hospital stay compared with patients who were not frail, which worsened with increasing frailty (16). In a meta-analysis of 3,817 patients with COVID-19, the pooled prevalence for CFS 1–3 was 34% (32–36%), CFS 4–9 was 65% (61–70%). Each 1-point increase in CFS was associated with 12% increase in mortality rate (17). A series of studies in this field have shown that older patients with COVID-19 have a higher incidence of frailty, and frailty has a significant negative effect on the prognosis of COVID-19 patients.

## Potential Pathogenesis

At present, the exact pathogenesis of frailty in old adults with COVID-19 has not been fully elucidated. A large number of studies have found that the levels of inflammatory factors are increased in both COVID-19 and frailty patients (18–21). Inflammatory response may be involved in the potential pathogenesis. During the acute phase of viral infection, circulating inflammatory cytokines are elevated in old patients with COVID-19. Upregulated Interleukin-6 (IL-6), activates STAT3 by tyrosine phosphorylation to trigger inflammatory response through the JAK/STAT signaling pathway (22). The resulting inflammation can generate Reactive Oxygen Species (ROS) causing both oxidative damage and eliciting an amplification of the cytokines' release, inducing a positive feedback loop resulting in a chronic systemic pro-inflammatory state where tissue injury and healing mechanisms proceed simultaneously (23). Besides, increased inflammation contributes to markers of blood clotting elevate, and long-term high coagulation state increases the risk of blood clots (24). High levels of inflammatory cytokines are also associated with a risk of muscle insufficiency, decreased skeletal muscle mass and loss of strength, leading to sarcopenia (25). These accumulative impairments can lead to a decline in multi-system function in elderly patients with COVID-19, which we called frailty.

**Figure 1:**
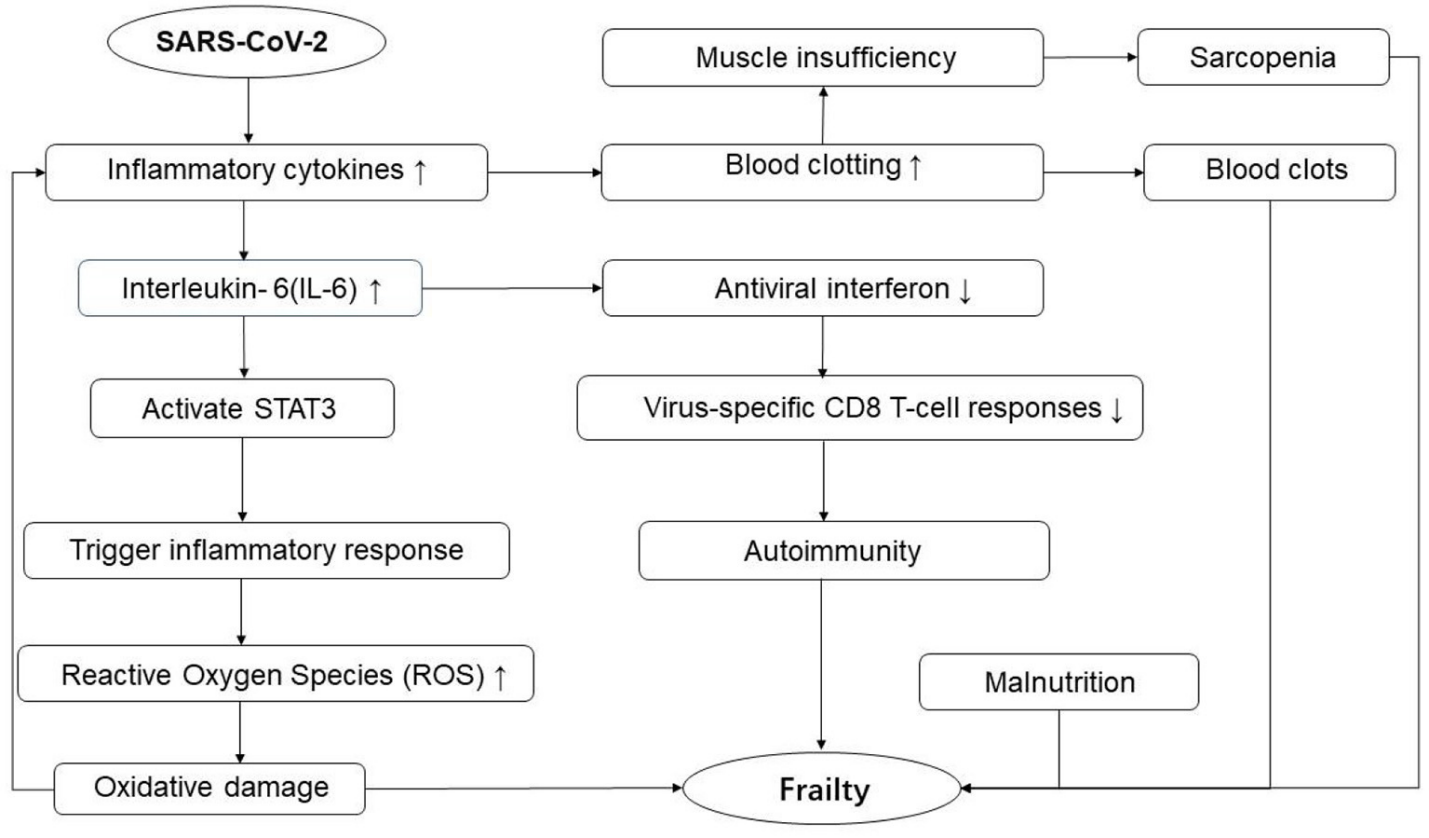
Potential pathogenesis of frailty in elderly patients with COVID-19.

Interleukin-6 also has a broad effect on cells of the immune system. It can induce the transcription factor Bcl-6 to transform naive T cells into follicular helper T cells, helping to initiate the adaptive immune response and to regulate auto-tolerance, to prevent autoimmunity (26, 27). However, high levels of IL-6 in old patients with COVID-19 can inhibit the response of alveolar macrophages and dendritic cells to influenza virus to produce antiviral interferon. It can further inhibit virus-specific CD8 T-cell responses (28, 29), and immune repertoire would be skewed to the memory phenotype, T cell clonal expansion, increased autoimmune antibody production that leads to frailty (30) (Figure 1). Meanwhile, because of suppressed virus-specific CD8 T-cell responses, it is difficult to clear coronavirus. Thus, frailty interacts with COVID-19 in a vicious cycle, and early identification of COVID-19 combined with frailty and timely intervention measures would significantly improve the quality of life and improve prognosis.

Malnutrition plays a key role in the pathogenesis of both frailty and sarcopenia. Elderly patients with COVID-19 often show low intake during hospitalization, forming an acute negative nutritional balance, which is characterized by the deficiency of multiple nutrients. If there are insufficient proteins and lipids to keep organ function and muscle activity, muscle will be catabolized to provide energy leading to sarcopenia (31). If old COVID-19 patients are lack of 25-hydroxyvitamin D, they will develop osteoporosis, and are susceptible to systemic infection, damage immune response, and even autoimmunity (32), promoting the occurrence of frailty. Meanwhile, with the appearance of frailty, the appetite would slough to aggravate malnutrition (33), which is not conductive to the prognosis of COVID-19. Therefore, early nutritional intake of elderly patients with COVID-19 is of great significance to prevent frailty.

## Post-COVID-19 Implications

The old COVID-19 persons combined with frailty tend to experience substantially more severe symptoms and lethality. Infection produces biological damage and loss of homeostasis. This ultimately contributes to accelerated aging and the development of age-related diseases (34).

About the impact of infections on physical function, only three studies performed objective physical and motor function tests, which targeted different physical domains (35–37). Despite the methodological heterogeneity, most studies support the hypothesis that infections impair physical function. The potential mechanisms are multiple. Prolonged immobility during hospitalization accentuates muscle-atrophy and levels of proinflammatory cytokines, which further lead to muscle loss and sarcopenia (38, 39). Decreased caloric and nutritional intake is a common feature of infectious diseases; this can negatively affect muscle health and function.

About the impact of infections on cognitive function, many studies supported the possibility that pneumonia, and virus infection to a lesser extent, negatively affect cognition. The neurotrophic propensity of the virus and the sustained pro-inflammatory status induced by the infection may trigger or accelerate neurodegenerative processes through increased deposition of beta-amyloid and microglia activation (35, 40–42). Similarly, the systemic inflammatory status and the boost in the oxidative processes might explain the impact of pneumonia on cognition. Further, pneumonia-related hypoxia is also linked to neurodegeneration and cerebrovascular lesions, which has been shown to predispose individuals to cognitive impairment and dementia (43).

## Evaluation Method

During the pandemic, an appropriate evaluation method for early identification of elderly COVID-19 patients who are prone to frailty is needed. Early intervention measures and reasonable allocation of medical resources are conducive to improving the prognosis. However, so far, there is no standard evaluation method for COVID-19 with frailty. Frequently used frailty evaluation methods recommended by the International Conference of Frailty and Sarcopenia Research (ICFSR) in 2019 include the following (discussed later and summarized in Table 1).

**Table 1:** Performance of different evaluation methods to identify frailty among patients with COVID-19.

	**Cutoffs**	**Strengths**	**Weaknesses**
CFS	Score according to considered variables, in general frail if CFS score >4	Simple; no physical testing required; score associated with mortality	Scoring somewhat subjective
FI	Ratio of accumulated deficits and considered variables, in general frail if FI >0.25	Meaningful results in all patients; irrespective from age and disability	Time-consuming; need for comprehensive geriatric assessment
FP	Five pre-defined criteria to categorize a patient as robust (none of the criteria), pre-frail (one or two criteria), and frail (three or more criteria)	Possible before comprehensive geriatric assessment; pre-clinical evaluation	Not applicable to disabled older patients
FRAIL	Five pre-defined criteria to categorize a patient as robust (one or none of the criteria), pre-frail (two criteria), and frailty (three or more criteria)	Simple; cheap; no physical testing required	Scoring somewhat subjective
EFS	Score according to considered variables, in general frail if EFS score>6	Good poly-dimension, credibility, and internal consistency; no physical testing required; a reliable predictor for mortality	Scoring somewhat complicated

### Clinical Frailty Scale

In 2020, the National Institute for Health and Care Excellence (NICE) published a quick guidance on the care of patients with COVID-19, which recommended to access patients aged 65 and over with CFS. Clinical frailty scale is a simple and inexpensive frailty scale that scores patients based on their daily function (44). A lot of studies revealed that the CFS has been widely used in multiple settings. The association of CFS score with clinical outcomes highlights its utility in the care of the aging population (45). Labenz et al. (46) believed that CFS is independent of comorbidities or age, and regular testing with CFS at hospital admission may identify patients at higher risk for worse disease progression and improve the prognosis for COVID-19 patients. Sablerolles et al. (47) believed that CFS score is a suitable risk marker for hospital mortality in patients with COVID-19 over 65 years. Frail patients (CFS 6–9) of all ages admitted with COVID-19 have a significantly higher hospital mortality than fit patients (CFS 1–3). But there is no significant difference in hospital mortality between mildly frail patients (CFS 4–5) and fits patients who were admitted to intensive care. Therefore, CFS is an appropriate scale to assess frailty in elderly patients with COVID-19, and patients with CFS 6–9 points should be given more medical resources and a higher level of care.

### Frailty Index

The calculation method of FI is the ratio of the number of defects existing in patients to the total number of defects designed, including social characteristics, clinical signs, symptoms, comorbidities, laboratory examination, and imaging examinations. These items can be adjusted according to individual differences. Kojima et al. (48, 49) believed that FI is a good indicator of mortality risk for all patients, irrespective from age and disability. Moreover, FI can evaluate frailty status in a graded manner to make a more precise risk prediction, rather than just three frailty categorization by frailty phenotype (robust, pre-frail, and frail). However, the evaluation method of FI is time-consuming and needs comprehensive geriatric assessment (49). The establishment of electronic FI simplifies the use of FI, and Clegg et al. demonstrated that electronic FI is a reliable predictor for mortality (50). With the popularization of electronic medical records in hospitals, the use of electronic FI to predict mortality in elderly patients with COVID-19 is considered convenient and feasible (51).

### Frailty Phenotype

Frailty phenotype, developed and verified by Fried et al. in 2001, is a commonly used evaluation method to identify frailty or pre-frailty in current studies (5). Subjects were diagnosed as frailty if three or more of the following five criteria are met: decreased walking speed, decreased grip strength, decreased physical activity, fatigue, and unexplained weight loss. Fried et al. demonstrated that FP provides a potential basis for clinical assessment for those who are frail or at risk, which could be used for pre-clinical evaluation (49), and for future research to develop interventions for frailty based on a standardized ascertainment of frailty (6). Cesari et al. FP is deemed to have a high relation with nutritional status (52), which may be more suitable for the evaluation of elderly COVID-19 patients with anorexia. It is also more reasonable to give nutritional supplements to patients based on FP. Petermann-Rocha et al. (53) found that there is no significant difference between FP and FI in evaluating COVID-19 patients combined with frailty. However, FP is not applicable to disabled older patients for that it requires objective measurement of stride speed and grip strength (49).

### The Frail Scale

As FP is not applicable for patients with impaired physical function or patients in the acute phase whose grip strength and gait speed can't be accurately measured, The FRAIL scale was provided by the International Society of Nutrition and Aging. The assessment of FRAIL is similar to FP. However, the assessment of FP is based entirely on patients themselves, without any measurement tools. Aprahamian et al. proved that FRAIL can be used as a screening instrument for frailty (time and cost-effective) (54). As a simple and easy scale, it has been adopted by many countries and regions (55). May et al. (56) conducted a prospective cohort study of 114 elderly COVID-19 patients to examine the association between frailty and COVID-19, and it was demonstrated that the FRAIL scale can help to predict the risk of frailty in elderly COVID-19 patients at an early stage. Clinical work is busy, and frailty assessment for elderly patients is labor- and time-consuming. FRAIL is of high sensitivity and convenience, but accuracy is insufficient for that evaluation methods based on the patient's description alone. As a result, it can be used for preliminary screening of frail patients to improve clinical work efficiency.

### Edmonton Frailty Scale

Compared with the above four evaluation methods, EFS covers a wider range of assessment items, such as nutrition, mood, and medication, which has good poly-dimension, credibility, and internal consistency. Perna et al. (57) proved that EFS could not only reflect the frailty risk of patients reliably, but also reveal the reason for frailty. Similarly, EFS has an important relationship with independent evaluation scales in various fields. Chan et al. (58) found an important association between EFS and the Geriatric Depression Scale (GDS). Faria et al. (59) found an important association between EFS and the Mini-Mental State Examination (MMSE). Izawa et al. (60) found an important association between EFS and the Mini-Nutritional Assessment (MNA). Therefore, providing EFS for elderly patients with COVID-19 and selecting appropriate independent assessment scale according to EFS to give etiological therapy may be beneficial to improve the prognosis of old COVID-19 patients. At present, there is a lack of relevant literature on evaluating elderly COVID-19 patients with EFS, which needs to be studied urgently.

## Intervention Measure

Frailty is a dynamic and manageable process that can be prevented, delayed or even reversed with interventions and health strategies. At present, there is no recognized intervention measures for COVID-19 with frailty. All reviews suggested the adoption of regular physical exercise and nutrition support (34, 61, 62).

### Physical Exercise

As people get older, their motion decreases and physical function declines, increasing the risk of frailty. The American College of Sports Medicine (ACSM) recommends multicomponent exercise, including resistance exercise, aerobic exercise, and coordination and balance exercises, as the best physical intervention for preventing and improving frailty.

First of all, sarcopenia, as a common potential factor in frailty, is characteristic of progressive and general loss of skeletal muscle mass and strength with a risk of adverse outcomes such as physical disability, poor quality of life and death (63). Resistance exercise can improve muscle quality and function by damaging the ultrastructural of muscle, releasing inflammatory cytokines and growth factors, and increasing protein synthesis (64). One meta-analysis found that intensities ranging from 70 to 95% of 1-RM significantly increase muscle strength, but decreasing the intensity of the weights to 30–40% of 1-RM during the first 2 weeks of the program to reduce muscle soreness may be more reasonable (65). Besides, resistance exercise also exerts a mechanical load on bones consequently leading to increase in the bone strength so as to reduce the incidence of osteoporosis (66). Secondly, aerobic exercise (such as swimming, cycling, and walking) is also indispensable. Aerobic exercise ensures adequate oxygen supply and transport of nutrients and metabolites and maintains the normal functioning of tissues and organs. It is beneficial to increase brain blood flow in old people, nourish nerve, and help improve cognitive function (67). Finally, fall is a common problem for older people, and coordination and balance exercises can help old people adjust their posture. At the same time, resistance exercise can strengthen the skeletal system, and aerobic exercise can improve the nervous system, which can all prevent patients from fall (68). A random controlled experiment showed that there are 29–60% proportional reductions in the incidence, duration, and overall severity of acute respiratory infection in the group in which patients insist on physical exercise at a moderate intensity (69). More and more researchers believed that physical exercise can enhance the immune response and mitochondrial antiviral ability to improve the prognosis of COVID-19 (70). Meanwhile, it is necessary for elderly COVID-19 patients to do physical exercise at home to help control COVID-19 transmission during a pandemic (71).

### Nutrition Support

As the main contributor of muscle anabolism in the elderly, protein is an indispensable energy substance. A long-term study suggested that protein supplementation for 24 weeks (15 g of milk protein daily at breakfast and lunch), coupled with resistance training, can improve muscle mass, strength, and physical function (72). More protein intake does not mean healthier, and excessive intake may increase the risk of frailty (73). The distribution of protein intake is more important than the total protein intake, and frailty patients tend to consume more protein at noon, and less in the morning (74). Supplementing protein according to the consumption may be much conducive to the prevention and relief of frailty. At the same time, the source of protein is important, and plant protein and soy protein are better for lowering LDL-c and protecting the cardiovascular system (75).

The endogenous antioxidant function of the elderly decreases with age, and the sensitivity to oxidative stress increases. Oxidative stress can accelerate the generation of osteoclasts and the apoptosis of osteoblasts, which does not only lead to osteoporosis, but also impair heart and brain function, and increase the risk of frailty in old people (76). Carotenoids from fruits and vegetables have obvious antioxidant effect and reduce oxidative stress by destroying the formation of oxygen free radicals. It can also reduce the inflammatory cytokines induced by Aβ42 and inhibit the inflammatory response which could be beneficial in inhibiting the inflammatory progression of COVID-19 (77). It also plays an important role in the cognitive impairment. Carotenoids have a high binding force with Alzheimer's disease-related receptors (histone deacetylase and P53 kinase receptors) (78), which could become antagonists of Alzheimer's disease. According to relevant studies, long-term carotenoid supplementation at a dose of 10 mg/day is safe and effective. Excessive consumption of carotenoids increases the risk of lung cancer that is not conducive to the prognosis of COVID-19 (79, 80).

Elderly people are chronically low in calories. Schoufour et al. observed that with every 418·4 kJ (100 kcal) increase, the odds to be frail are 5% lower (81). But there is no evidence to support the benefits of a high-calorie diet. Kim et al. (82) suggested that maintaining a daily energy supplement of 400 Kcal and 25 g of protein can significantly improve physical fitness and prognosis of elderly patients with COVID-19.

Low levels of vitamins (such as A, D, E, B6, and B12) and minerals (such as calcium, zinc, and selenium) are found to be independent risk factors for frailty, which play important roles in inhibiting inflammation, reducing oxidative stress, maintaining muscle function, improving osteoporosis, and preventing falling. Research showed the benefits on balance and muscle strength with daily doses of 800 IU or more of vitamin D (83).

In addition, a reasonable diet structure is much conducive to the absorption of nutrients. Mediterranean diet is generally considered as a reasonable dietary structure, which is characterized by more intake of fresh fruits, vegetables, and high-quality protein, less intake of red meat and dairy, and moderate intake of drinking, mainly with wine. Multiple studies showed that the Mediterranean diet is beneficial for prognosis of old frail people (84).

### Medical Intervention

It has been suggested that defects in multiple hormonal systems are potential factors in the frailty (85). The main endocrine hormone suitable for the medical intervention of frailty include sex hormone, growth hormone, and insulin-like growth factor (IGF). Androgen, including testosterone and dehydroepiandrosterone, has the function of maintaining musculoskeletal growth. In the elderly patients, the decreasing androgens make them lose muscle mass and strength, so that androgen replacement therapy could be beneficial for them. However, Nair et al. (86) found that daily androgen supplementation of 50 mg was not beneficial for muscles of elderly men, and increased the risk of cardiovascular and prostate adverse events. Progesterone is used primarily to stimulate appetite to improve absorption of nutrient, but some studies suggested that supplementation with progesterone after high-intensity resistance training seems to impair the effects of exercise, leading to decreased muscle mass, and function after exercise (87). Growth hormone plays an important role in the skeletal muscle growth. Insulin-like growth factor can regulate the production of a variety of transcription factors, which participates in inflammation and the expression of autophagy-related genes that are key mechanisms associated with frailty (88). However, there are few studies on the use of these hormones in old COVID-19 patients combined with frailty, and the efficacy and safety of them are still uncertain. More related studies are needed for further confirmation.

In addition to hormone, a 16-week treatment with metformin (500 mg twice daily) has been shown to improve walking speed in frail patients (85). Probiotic can improve the physical strength and grip strength of the elderly by regulating gastrointestinal flora (89). Supplementation with L-carnitine (1.5 g/day) for 10 weeks significantly improved grip strength in the elderly, and some subjects even changed from a pre-frailty state to a healthy state after intervention (90). However, the above conclusions are lack of sufficient evidences and need to be proved by further researches. At present, the ICFSR does not recommend medical intervention for frailty because its effectiveness is unclear, and it is not possible to assess whether the benefits of medical intervention outweigh the adverse consequences (91).

## Conclusion

A series of studies suggested that elderly COVID-19 patients have a high incidence of frailty, and frailty is detrimental to COVID-19 prognosis. Due to the interaction between frailty and COVID-19, a vicious cycle leads to the poor prognosis. At present, there is no generally accepted consensuses for the evaluation and intervention of frailty in elderly patients with COVID-19, and there is not enough clinical understanding of it. Therefore, it is of great significance to improve the awareness of clinicians on the importance of early recognition and intervention methods. In clinical work, the elderly COVID-19 patients should be evaluated as early as possible, and multi-component physical exercise including resistance exercise, aerobic exercise, and coordination and balance exercises should be recommended, following the Mediterranean diet and avoiding mindless drug use.

## Author Contributions

All authors listed have made a substantial, direct and intellectual contribution to the work, and approved it for publication.

## Conflict of Interest

The authors declare that the research was conducted in the absence of any commercial or financial relationships that could be construed as a potential conflict of interest.
